# Discovery of a Novel Target for the Dysglycemic Chromogranin A Fragment Pancreastatin: Interaction with the Chaperone GRP78 to Influence Metabolism

**DOI:** 10.1371/journal.pone.0084132

**Published:** 2014-01-20

**Authors:** Nilima Biswas, Ryan S. Friese, Jiaur R. Gayen, Gautam Bandyopadhyay, Sushil K. Mahata, Daniel T. O'Connor

**Affiliations:** 1 Departments of Medicine, University of California San Diego, La Jolla, California, United States of America; 2 Department of Pharmacology, University of California San Diego, La Jolla, California, United States of America; 3 Institute for Genomic Medicine, University of California San Diego, La Jolla, California, United States of America; 4 VA San Diego Healthcare System, San Diego, California, United States of America; University of Minnesota, United States of America

## Abstract

**Rationale:**

The chromogranin A-derived peptide pancreastatin (PST) is a dysglycemic, counter-regulatory peptide for insulin action, especially in liver. Although previous evidence for a PST binding protein has been reported, such a receptor has not been identified or sequenced.

**Methods and Results:**

We used ligand affinity to purify the PST target, with biotinylated human PST (hCHGA_273–301_-amide) as “bait” and mouse liver homogenate as “prey”, and identified GRP78 (a.k.a. “78 kDa Glucose Regulated Protein”, HSPA5, BIP) as a major interacting partner of PST. GRP78 belongs to the family of heat shock proteins (chaperones), involved in several cellular processes including protein folding and glucose metabolism. We analyzed expression of GRP78 in the absence of PST in a mouse knockout model lacking its precursor CHGA: hepatic transcriptome data revealed global over-expression of not only GRP78 but also other heat shock transcripts (of the “adaptive UPR”) in CHGA(−/−) mice compared to wild-type (+/+). By contrast, we found a global decline in expression of hepatic pro-apoptotic transcripts in CHGA(−/−) mice. GRP78's ATPase enzymatic activity was dose-dependently inhibited by PST (IC_50_∼5.2 µM). PST also inhibited the up-regulation of GRP78 expression during UPR activation (by tunicamycin) in hepatocytes. PST inhibited insulin-stimulated glucose uptake in adipocytes, and increased hepatic expression of G6Pase (the final step in gluconeogenesis/glycogenolysis). In hepatocytes not only PST but also other GRP78-ATPase inhibitors (VER-155008 or ADP) increased G6Pase expression. GRP78 over-expression inhibited G6Pase expression in hepatocytes, with partial restoration by GRP78-ATPase inhibitors PST, VER-155008, or ADP.

**Conclusions:**

Our results indicate that an unexpected major hepatic target of PST is the adaptive UPR chaperone GRP78. PST not only binds to GRP78 (in pH-dependent fashion), but also inhibits GRP78's ATPase enzymatic activity, and impairs its biosynthetic response to UPR activation. PST decreases insulin-stimulated cellular glucose uptake, and PST as well as other chaperone ATPase activity inhibitors augment expression of G6Pase; GRP78 over-expression antagonizes this PST action. Analysis of the novel PST/GRP78 interaction may provide a new avenue of investigation into cellular glycemic control as well as dysglycemia.

## Introduction

Pancreastatin (PST, human CHGA_250–301_-amide) is derived from the proteolytic cleavage of its precursor, chromogranin A (CHGA), an acidic glycoprotein abundant in secretory granules of chromaffin cells and also present throughout the neuroendocrine system [1]. Several processing intermediates have been reported for PST in cells or tissues, and all contained the biologically conserved active carboxyl-terminal part of the molecule [2]–[5]. The amidated carboxyl terminus of PST is a feature in common with many neuropeptides and gastrointestinal hormones such as NPY and PYY.

The activity of PST as a regulatory gastroenteropancreatic peptide has been established in the light of a variety of biological effects in a number of tissues [6]–[9], including its role in modulation of energy metabolism, with a general counter-regulatory effect to insulin. PST induces glycogenolysis in liver and lipolysis in adipocytes [10], [11]. PST inhibits insulin action in rat adipocytes in a dose dependent manner and within a physiological range of concentration [12] and inhibits both basal and insulin-stimulated glycogen synthesis [13]. Metabolic effects of PST have been confirmed in humans and naturally occurring human variants have been found, such as Gly297Ser in the functionally important carboxyl terminus of the peptide, substantially increasing its potency to inhibit cellular glucose uptake [14].

Preliminary pharmacological characterization of a PST binding protein has been described in rat liver, hepatoma, adipocytes and heart membranes [15]–[18]. A putative PST receptor was purified from rat liver membranes by Sanchez-Margalet et al., and may be physically associated with a Gαq/11 protein [19]; however the final identification and sequencing of such a PST receptor have been elusive so far. PST seems to activate a receptor signaling pathway that is typically associated with seven-spanning transmembrane receptors coupled to Gq-PLCβ-calcium-PKC signaling and mediated through protein kinase C and NO-dependent pathways [20]–[23]. Increased PST plasma levels, correlating with catecholamine levels have been found in insulin resistant states such as gestational diabetes, essential hypertension or type 2 diabetes mellitus [24]–[27].

In studies by Gayen et al, CHGA knockout (−/−) mice display *enhanced* glycemic control in spite of *low* plasma insulin levels, because of increased liver insulin sensitivity, and treatment of such mice with PST increased blood glucose in association with augmented phosphoenolpyruvate carboxykinase and glucose-6-phosphatase mRNA expression [23]. The pathway from PST towards these effects may include IRS1/2-phosphatidylinositol 3-kinase-AKT-FOXO-1 activation, as well as effects on insulin-induced maturation of SREBP1c by PKC and elevated NO [23].

To understand the earliest stage of PST action, we searched for its cellular interacting partner (or target) by PST-ligand (“bait”) affinity chromatography on liver proteins (“prey”). We found that PST interacts directly with the chaperone GRP78, a widely expressed, ∼78-kDa “Glucose-Regulated Protein”, also known as “Binding Immunoglobulin Protein” (BIP) or Heat Shock Protein A5 (HSPA5) [28]. The interaction of GRP78 with PST, and its implications for glucose homeostasis and dysglycemia are explored here.

## Experimental Procedures

### Ethics statement

The experimental animal (mouse) studies were approved by the UCSD Institutional Animal Care And Use Committee (Protocol S00048M).

### Ligand affinity isolation of a PST binding protein

Freshly obtained normal mouse liver was homogenized in cold, freshly prepared buffer (10 mM Hepes, pH 7.4, 0.1 mM EDTA) with a cocktail of protease inhibitors (Protease inhibitor cocktail set III, Calbiochem at 1∶100: PMSF 5 mM, benzamidine hydrochloride 50 mg/mL, TLCK [N-Tosyl-Lys Chloromethyl Ketone] hydrochloride 0.1 mM), and centrifuged at 50,000 g for 30 min to pellet crude membranes. The membrane preparation was washed once with the same buffer and then solubilized with 1% v/v Triton X-100 in 25 mM Hepes pH 7.4, 100 mM NaCl, 2 mM MgCl_2_, 1 mM KCl and protease inhibitor cocktail as mentioned above, for 1 hr at 4°C, then centrifuged at 100,000 g for 1 hr, and the supernatant was saved.

Biotinylated human PST (hCHGA_273–301_-amide) was synthesized by placing a biotin residue at the amino terminus of the peptide, separated by a spacer consisting of four amino acids (EAQD) from the natural sequence: Biotin-EAQD-PEGKGEQEHSQQKEEEEEMAVVPQGLFRG-amide. A ligand affinity column was prepared by incubating 5 mg of biotin-PST-amide with 1 ml of 50% streptavidin agarose resin (Thermo Scientific) in column buffer (25 mM Hepes, pH 7.4, 100 mM NaCl, 2 mM MgCl_2_, 1 mM KCl, 1% Triton and protease inhibitor cocktail) for 1 hr at 4°C.

The streptavidin resin was washed extensively with the same buffer and then incubated with solubilized liver membranes for 18 hr at 4°C. The resin was packed into a chromatography column, washed with the column buffer containing 0.1% Triton, and bound proteins were eluted with column buffer adjusted down from pH 7.4 to 5.5. Fractions eluted were concentrated by TCA precipitation and analyzed on 8–16% SDS-PAGE gradient slab gels, stained with coomassie blue G-250 (SimplyBlue Safestain; Invitrogen). The resulting ∼75 kDa major protein band was excised, subjected to in-gel digestion with trypsin (see below), and the resulting peptides were separated by reverse-phase liquid chromatography followed by tandem mass spectrometry.

During the original experiment, a minor band of ∼175 kDa was also observed, but not characterized further. A similar/control ligand affinity experiment on liver membranes was performed with the CHGA-derived biologically active peptide catestatin (human CHGA_352–372_) as the immobilized biotinylated ligand.

### In-gel digest of the PST-binding protein

Gel slices were cut to 1×1 mm cubes and destained 3 times by first washing with 100 µl of 100 mM ammonium bicarbonate for 15 min, followed by addition of 100 µl acetonitrile (ACN) for 15 min. The gel pieces were then dried in a SpeedVac and reduced by mixing with 200 µl of 100 mM ammonium bicarbonate-10 mM DTT by incubating at 56°C for 30 min. The liquid was removed and 200 µl of 100 mM ammonium bicarbonate/55 mM iodoacetamide was added to gel pieces and incubated at room temperature in the dark for 20 min. After the removal of the supernatant and one wash with 100 mM ammonium bicarbonate for 15 min, the same volume (200 µl) of ACN was added to dehydrate the gel pieces. The solution was then removed and samples were vacuum-dried (SpeedVac). For digestion, enough solution of ice-cold trypsin (0.01 µg/µl) in 50 mM ammonium bicarbonate was added to cover the gel pieces and set on ice for 30 min. After complete rehydration, excess trypsin solution was removed, replaced with fresh 50 mM ammonium bicarbonate, and left overnight at 37°C. The peptides were extracted twice by addition of 50 µl of 0.2% formic acid and 50% ACN, and vortex-mixed at room temperature for 30 min. The combined extractions were analyzed directly by reverse-phase liquid chromatography (LC) in combination with tandem mass spectroscopy (MS/MS) using electrospray ionization [29].

### LC-tandem-MS/MS analysis

Proteins extracted from SDS-PAGE gels and trypsin-digested were analyzed by liquid chromatography (LC, C-18)-tandem-MS/MS with electrospray ionization. Nanospray ionization experiments were performed with a QSTAR-Elite hybrid mass spectrometer (AB/MDS Sciex) interfaced to a nanoscale reverse-phase high-pressure liquid chromatograph (Tempo) with a 10 cm-180 micron ID glass capillary packed with 5-µm C-18 Zorbax™ beads (Agilent). The buffer compositions were as follows. Buffer A was composed of H_2_O containing 2% ACN, 0.2% formic acid, and 0.005% TFA; buffer B was composed of 100% ACN containing 0.2% formic acid, and 0.005% TFA. Peptides were eluted from the C-18 column into the mass spectrometer using a linear gradient of 5–60% buffer B over 60 min at 400 µl/min. LC-MS/MS data were acquired in peak-dependent fashion by selecting the 4 most intense peaks with charge state of +2 to +4 that exceeds 20 counts, with exclusion of former target ions set to “360 sec” and mass tolerance for exclusion set to 100 ppm. Time-of-Flight (TOF) MS data were acquired at *m/z* 400 to 1600 Da, while MS/MS data were acquired from *m/z* 50 to 2,000 Da. Peptide identifications were made using the Paragon algorithm executed in Protein Pilot 2.0 (Life Technologies).

### Plasmids

The human G6P-ase promoter-luciferase reporter plasmid has been described [23]. The GRP78 (BIP) promoter-luciferase reporter was a gift from Bruce M. Spiegelman (Harvard Medical School). The pCMV promoter-driven human GRP78 (HSPA5) expression plasmid was from Origene <www.origene.com/>. pSV-Beta-Gal (a transfection efficiency control plasmid, in which the SV40 early promoter drives expression of beta-galactosidase) was obtained from Promega <www.promega.com>.

### GRP78's ATPase enzymatic activity

Concentration-dependent spectrophotometric assays were carried out using recombinant human GRP78 protein (catalog # ab78432, Abcam Inc., Cambridge, MA) at concentrations of 0.063, 0.125, 0.25 and 0.5 µM in 50 µl of assay buffer (20 mM Tris, pH 7.5, 50 mM KCl, 1.5 mM MgCl_2_). The reaction was started by adding 100 µM ATP and incubated for 37**°**C for 30 min. Liberated free phosphate (Pi) was measured by a Malachite green-phosphate assay (catalog #10009325, Cayman <https://www.caymanchem.com>). The assay method is based on formation of a complex between malachite green molybdate and Pi (free orthophosphate) that absorbs at 620–640 nm. Assays to determine inhibition of ATPase activity were done with GRP78 (0.25 µM), with or without PST (0 to 10 µM).

### Transfection and luciferase reporter activity of human HepG2 hepatocytes

Cells were plated on 24 well tissue culture plates the day before transfection. 0.5 to 1.0 µg of total DNA per well was used for transfection with Transfectin (BioRad). In some experiments, pSV-Beta-Gal (Promega; in which the SV40 early promoter drives expression of beta-galactosidase) was co-transfected as a transfection efficiency control. After 5 hrs supernatants were removed and fresh media containing different compounds as mentioned in the respective figures were added. 18 hrs after transfection, supernatants were removed and cells were lysed in passive lysis buffer (Promega) and the luciferase and beta-galactosidase activity were measured. Luciferase activity was normalized by beta-galactosidase as well as cellular protein, determined by dye-binding (coomassie G-250; BioRad).

### Transcriptome of liver and adrenal gland in organisms with (CHGA[+/+]) or without (CHGA[−/−]) a functional CHGA gene

Genome-wide transcriptome profiling in CHGA knockout (−/−, CHGA KO) and wild-type (+/+, WT) mice (n = 3 biological replicates each) was measured in mRNA from the adrenal gland or liver, by Affymetrix GeneChip hybridization (MG-U74Av2 GeneChips for adrenal glands; Mouse 430A 2.0 GeneChips for livers), as described [30]. Pathway analyses, by functional clustering, were conducted with the NCI-DAVID (**D**atabase for **A**nnotation, **V**isualization and **I**ntegrated **D**iscovery) package, at <http://david.abcc.ncifcrf.gov/>. The DAVID algorithm addresses the redundant nature of genomic annotation that tends to dilute biological meaning during interpretation of gene expression data and is based on the hypothesis that similar functional annotations should have similar gene members. DAVID functional annotation clustering integrates kappa statistics and fuzzy heuristic clustering to group similar annotations into functional clusters. The P value associated with each annotation term is a modified Fisher exact score. The functional cluster enrichment score is the **−**log_10_ transformation of the geometric mean of all annotation terms within the functional cluster. Clusters are selected from Gene Ontology functional groups (GO at <http://amigo.geneontology.org>)

### 3T3-L1 pre-adipocytes: Culture and differentiation

3T3-L1 mouse pre-adipocytes from ZenBio <www.zen-bio.com> were cultured and differentiated to the adipocyte phenotype following the protocol from the manufacturer, in which adipocyte differentiation occurs in response to 3-isobutyl-1-methylxanthine (IBMX 500 µM), dexamethasone (1 µM), and insulin (1.7 µM).

### Measurement of cellular glucose uptake by adipocytes

Differentiated 3T3-L1 adipocytes cultured in 24-well plate were washed once with DMEM low glucose serum free medium and then serum-starved (serum free medium with 0.5% fatty acid-free BSA) for an additional 3 hrs, washed three times with Hepes/salts buffer (25 mM Hepes, pH 7.4, 120 mM NaCl, 5 mM KCl, 1.2 mM MgSO_4_, 1.3 mM CaCl_2_, 1.3 mM KH_2_PO_4_) and then incubated in the same buffer at 37**°**C. Subsequently the cells were stimulated with or without 100 nM insulin in the presence or absence of PST (100 nM) for 30 min, and 2-deoxy-D-[^3^H]glucose was added during the last 10 min at a final concentration of 100 µM (specific activity: 0.2 µCi/ml). Glucose uptake was terminated by washing three times with ice-cold PBS, and the cells were then lysed with 1 N NaOH for 30 min, neutralized and subjected to liquid scintillation counting.

### Statistics

Results are expressed as mean ± SEM. Inter-group comparisons were made by t test, 1-way or 2-way ANOVA, as appropriate, in Kaleidagraph <www.synergy.com>.

## Results

### PST binds liver target GRP78 (HSPA5, BIP) in pH-dependent fashion

**Figure 1:**
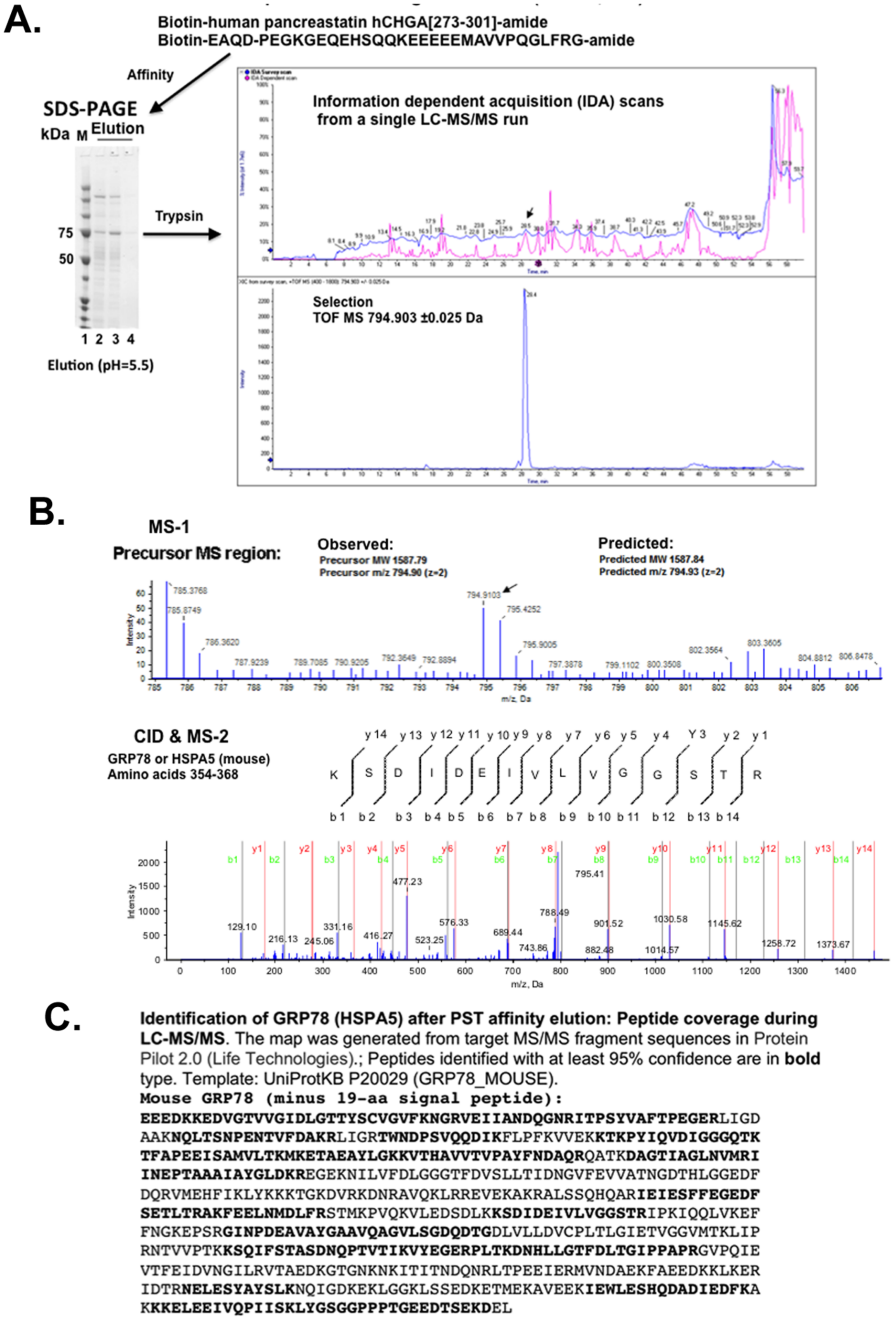
Identification of a PST binding protein from mouse liver membranes: Isolation and identification. (**1A**) **and** (**1B**) **Affinity chromatography followed by LC-tandem-MS/MS**. (**1A**): **SDS-PAGE separation, followed by trypsin digestion and C-18 liquid chromatography**. The mouse liver homogenate was subjected to ligand affinity chromatography using a biotinylated human pancreastatin (hCgA_273–301_-amide) peptide as “bait”. Triton-solubilized liver membranes (as “prey”) were incubated with bio-PST, which was pre-incubated with streptavidin agarose. Bound proteins were eluted by lowering pH to 5.5, and analyzed by SDS-PAGE. Gel lanes: 1 = Protein size standards; 2–4 = serial fractions during elution at lower pH = 5.5. The major ∼75 kDa band was excised from the gel and subjected to trypsin digestion, and the resulting peptides were separated by liquid chromatography (chromatogram shown above). Selection (arrow) of a peak for further analysis is also shown. (**1B**): **First and second dimensions of MS, with sequence identification**. The first dimension of MS is displayed above. In the second dimension of MS, one ion is chosen (arrow), and a representative MS/MS spectrum of mouse GRP78_354–368_ is shown. (**1C**): **Identification of GRP78 (HSPA5) after PST affinity elution: Peptide coverage during LC-MS/MS**. The map was generated from target MS/MS fragment sequences in Protein Pilot 2.0 (Life Technologies).; Peptides identified with at least 95% confidence are in **bold** type. Template: UniProtKB P20029 (GRP78_MOUSE).

To identify novel proteins that interact with PST, we used affinity chromatography with biotinylated human PST-amide and a murine liver homogenate, eluting the bound protein by lowering pH from 7.4 to 5.5, followed by analysis with LC-tandem-MS/MS mass spectrometry. The major protein band detected after coomassie blue staining had M_r_ ∼75 kDa (Fig. 1A). When subjected to trypsin digestion and LC-MS/MS analysis, the band was identified as GRP78 (BIP, HSPA5; Fig. 1, Table 1, Table S1), with 44 spectra spanning ∼66% of the GRP78 amino acid sequence (Fig. 1C). The target seemed to be full-length mouse GRP78 (rather than the cytosolic splice variant GRP78va [31]) since at the amino terminus the identified peptide extended up to (but not including) the 19-amino acid signal peptide (MMKFTVVAAALLLLGAVRA), while at the carboxy-terminus the identified peptides spanned KD of the KDEL retention signal.

**Table 1:** PST binding protein summary statistics.

Rank	% Coverage	Accession	Name	Peptides (>95% conf)
1	66.11	gi|254540168	78 kDa glucose-regulated protein precursor [Mus musculus]	44
2	60.09	gi|162461907	stress-70 protein, mitochondrial [Mus musculus]	47
3	59.75	gi|31981690	heat shock cognate 71 kDa protein [Mus musculus]	17
4	28.18	gi|31560705	long-chain-fatty-acid–CoA ligase 1 [Mus musculus]	11
5	53.25	gi|84781771	trypsin 10 [Mus musculus]	2
6	8.32	gi|126116585	keratin, type II cytoskeletal 1 [Mus musculus]	3
7	16.22	gi|112983636	keratin, type I cytoskeletal 10 [Mus musculus]	2
8	14.57	gi|71043961	trypsinogen 7 [Mus musculus]	3
9	5.78	gi|113195684	keratin, type II cytoskeletal 6B [Mus musculus]	2
10	26.42	gi|6755893	trypsin 4 [Mus musculus]	3
11	7.49	gi|239787090	peroxisomal acyl-coenzyme A oxidase 2 [Mus musculus]	1
12	22.03	gi|51092293	keratin, type II cytoskeletal 1b [Mus musculus]	2
13	12.80	gi|47059013	keratin, type II cytoskeletal 73 [Mus musculus]	2
14	24.39	gi|16716569	protease, serine, 1 [Mus musculus]	5
15	19.59	gi|114145561	keratin, type II cytoskeletal 8 [Mus musculus]	1
16	9.61	gi|22164776	keratin, type II cytoskeletal 79 [Mus musculus]	1
17	3.70	gi|172072677	urocanate hydratase [Mus musculus]	1
18	10.36	gi|7949055	hippocalcin-like protein 1 [Mus musculus]	1
19	9.39	gi|30424792	inactive hydroxysteroid dehydrogenase-like protein 1 [Mus musculus]	0
20	15.45	gi|6678439	anionic trypsin-2 precursor [Mus musculus]	2
21	5.74	gi|31981920	ftsJ methyltransferase domain-containing protein 1 [Mus musculus]	0
22	6.36	gi|124486710	DENN domain-containing protein 3 [Mus musculus]	0
23	10.59	gi|126157504	serine/arginine repetitive matrix protein 2 [Mus musculus]	0

The most abundant 23 proteins bound to/eluted from the PST affinity column are displayed, as well as the number of peptides identified in each protein. Peptides from the trypsin-digested SDS-PAGE protein band were analyzed by nano-LC-nano-ESI MS/MS, and then identified with Protein Pilot software.

Tryptic peptides from two other heat shock family proteins, stress 70 protein (GRP75, HSPA9) and heat shock cognate 71 kDa protein (HSPA8) were also identified, with overall rank order of abundance: 1>2>3 = GRP78>GRP75>HSPA8 (Table 1 & Table S1). In addition we noted trace quantities of typically observed peptides from trypsin and keratin.

Affinity chromatography with another CHGA derived peptide catestatin (human CHGA_352–372_) on liver homogenate did not pull down the GRP78 protein, as evidenced by the SDS-PAGE and subsequent LC-MS/MS analysis (results not shown).

### Liver and adrenal expression of endogenous GRP78, and other UPR components (adaptive and pro-apoptotic), in the absence of CHGA (i.e., CHGA[−/−] mice)

**Figure 2:**
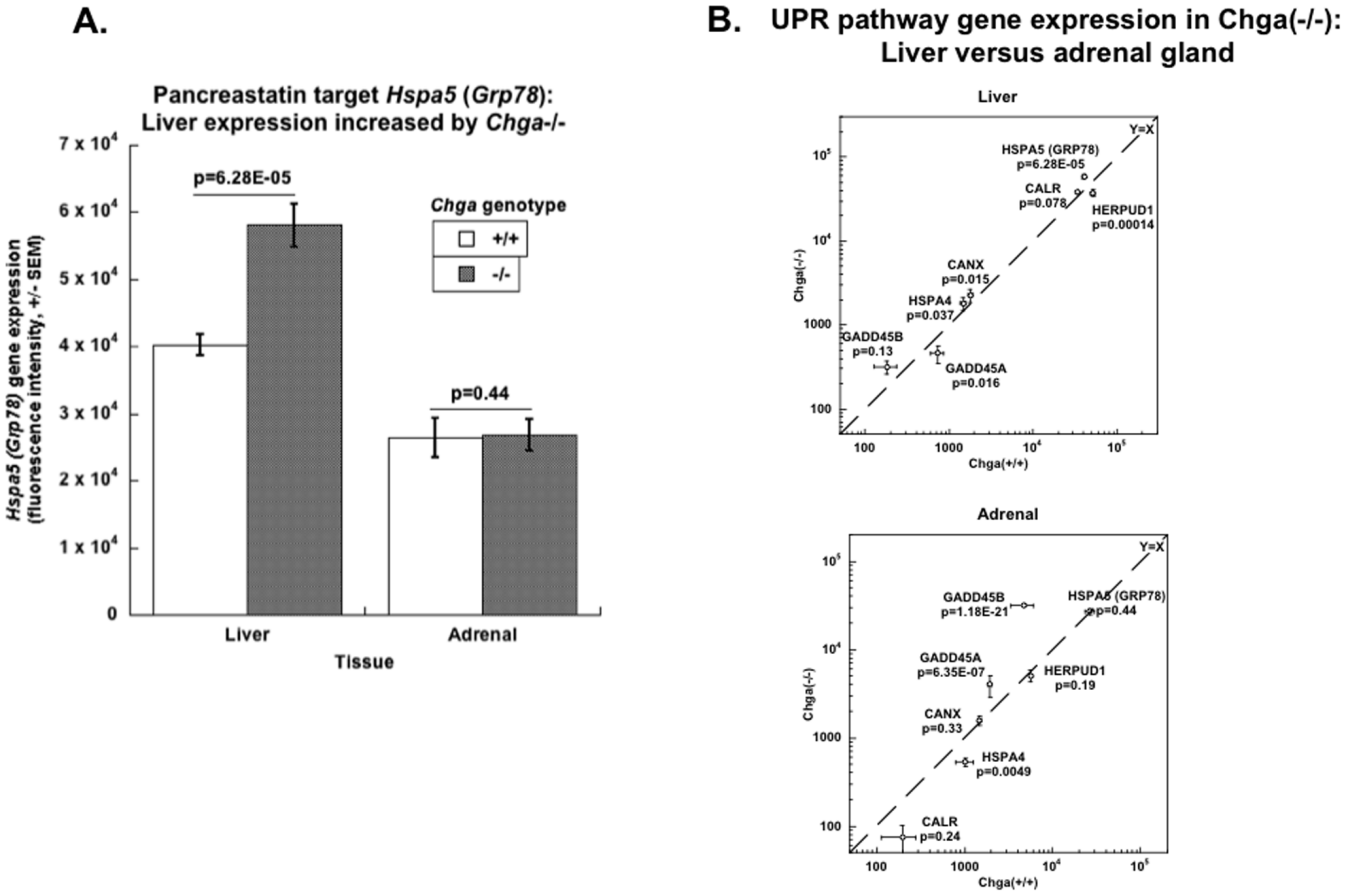
Expression of GRP78 and other UPR pathway genes in liver and adrenal transcriptomes of mice without CHGA (−/−). (**A**) **Liver and adrenal gland expression of GRP78** in CHGA(−/−) mice compared to wild-type (WT, +/+). (**B**) **Expression of UPR pathway genes** in liver (upper panel) and adrenal gland (lower panel).

The CHGA KO mouse displays *enhanced* glycemic control, with normal plasma glucose even in the face of reduced plasma insulin [23], [30]. To explore the role of CHGA as well as CHGA-derived PST, we profiled the liver transcriptome in CHGA +/+ versus −/− mice. Liver expression of GRP78 increased by +44% (−/− > +/+: p = 6.28E-05; 2 probes) in CHGA KO mice compared to wild-type (WT) (Fig. 2A), though not in adrenal gland.

Pathway analyses by the NCI-DAVID algorithms indicated global dysregulation of UPR pathway gene expression in liver of CHGA +/+ versus −/− mice. The peak pathway association was at Gene Ontology term GO:0006986 (“Response to unfolded protein”; genes: MM.378901, MM.31102, MM.390966, MM.4068, MM.271160, MM.24192, MM.43745, MM.10353, MM.247167, MM.440064, MM.18845, MM.2161, MM.341186, MM.29778, MM.387108, MM.28131, MM.56895, MM.29524, MM.289387, MM.110220, MM.142843, MM.260456, MM.29702, MM.378990, MM.275309, MM.273049, MM.55422, MM.39330, MM.1457, MM.149870, MM.4048, MM.340943, MM.28420, MM.24174, MM.1025, MM.293321, MM.294693, MM.27445, MM.29151, MM.20452, MM.299952, MM.217616, MM.337691, MM.42163, MM.12616, MM.169929, MM.391651, MM.21596, MM.240327, MM.653, MM.317701). The DAVID fold-enrichment score for the overall UPR pathway was 103.9 (p = 1.41E-130), with Bonferroni p = 7.98E-128, Benjamini p = 7.98E-128 (sic), and FDR = 2.07E-127. Selected UPR components are plotted in Fig. 2B.

**Figure 3:**
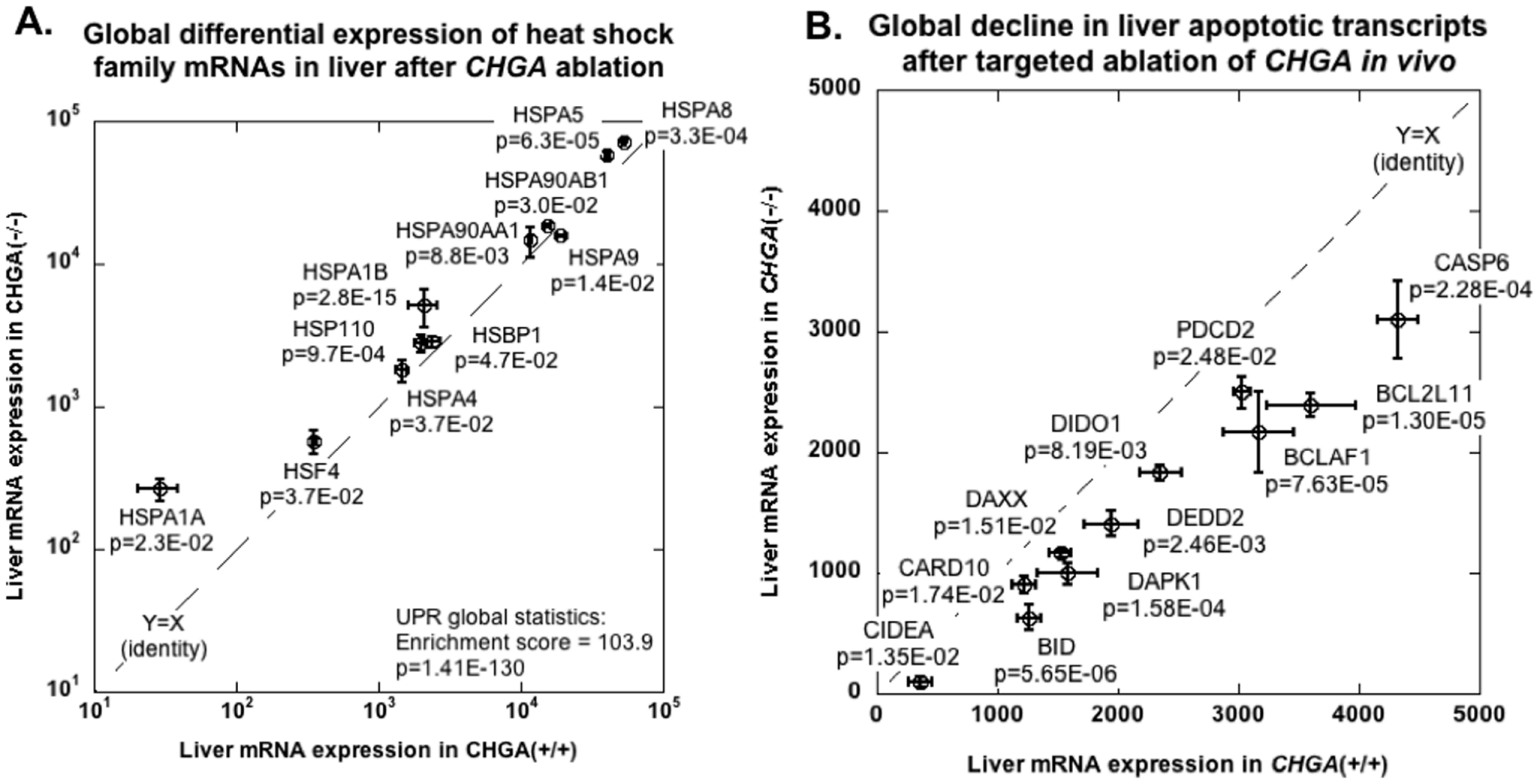
Expression of adaptive (heat shock family) and pro-apoptotic UPR genes in liver transcriptome of mice with systemic deletion of the CHGA gene. Transcripts were from the top 1000 most differentially expressed liver mRNAs (in −/− versus +/+ mice). (**A**) **Liver expression of heat shock family transcripts.** Each transcript was differentially expressed across the strains, with 10 of 11 transcripts *over*-expressed in −/− mice, and one under-expressed. Chi-square = 5.87, P = 0.0154. (**B**) **Liver expression of pro-apoptotic transcripts**. There was global *under*-expression of pro-apoptotic transcripts. Chi-square = 9.17, P = 0.0025.

Almost every heat shock family transcript (Figure 3A) on the chip was also significantly differentially expressed in mouse liver such that −/− > +/+: HSPA9 (−18%, p = 0.014), HSBP1 (+19%, p = 0.047), HSP90AB1 (+19%, p = 0.030; two probes), HSPA4 (+23%, p = 0.0.037), HSPA8 (+38%, p = 0.00033; 3 probes), HSP110 (+39%, p = 0.00097), HSP90AA1 (+25%, p = 0.0088; 2 probes), HSF4 (+63%, p = 0.037), HSPA1B (+147%, p = 2.80E-15; 3 probes), HSPA1A (+814%, p = 0.023).

Pro-apoptotic transcripts (Figure 3B) were globally expressed differentially in mouse liver, but this time as −/− > +/+: CASP6 (−28%, p = 0.00023), Bcl2l11 (−34%, p = 1.3000e-05), Bclaf1 (−32%, p = 7.6300e-05), BID (−50%, p = 5.6500e-06), Bcl2l1 (−44%, p = 0.04), Bcl2l2 (−58%, p = 0.04), Bcl2l10 (−82%, p = 0.04), Dido1 (−22%, p = 0.0082), DAXX (−24%, p = 0.015).

### PST unexpectedly *inhibits* the ATPase enzymatic activity of GRP78, as well its transcription

**Figure 4:**
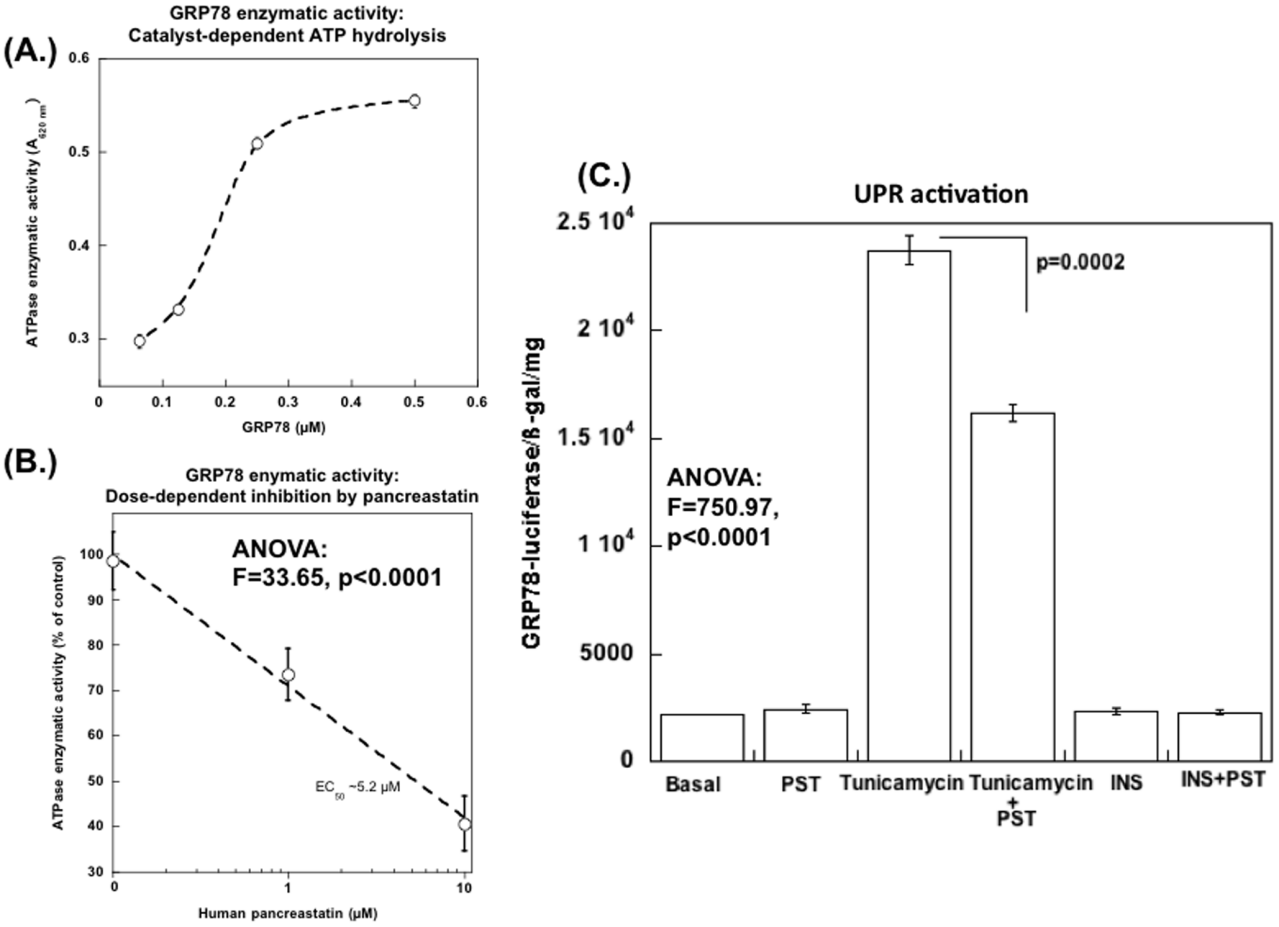
Pancreastatin *inhibits* the ATPase enzymatic activity of GRP78, as well as its transcription. (**A**) **ATPase enzymatic activity of GRP78** (concentration-dependent, steady-state) was measured as an increase in A_620_ (liberated as free phosphate, Pi) using 0.063, 0.125, 0.25 or 0.5 µM GRP78 in 50 µl of assay buffer, as described in experimental procedures. (**B**) **PST** ***inhibition*** **of the ATPase enzymatic activity of GRP78**. ATPase activity was measured in the presence of 0, 1, or 10 µM PST: IC_50_ of PST inhibition (by interpolation). (**C**) **GRP78 gene expression:** ***Inhibition*** **by PST**. Human HepG2 hepatocytes were transfected with GRP78 promoter driving a luciferase reporter along with beta Gal reporter plasmid and 5 hrs after transfection cells were treated with tunicamycin (5 µg/mL) in the presence or absence of PST (100 nM). Cells were harvested after 18–20 hrs for measurement of luciferase activity, beta gal activity and protein.

ATP binding and hydrolysis are essential for the chaperone activity of HSP70-family proteins; thus the UPR-adaptive effect of GRP78 is dependent on its functional ATP binding domain. HSP70s bind ATP with high affinity, and their ATPase activity, stimulated by binding to the unfolded protein, catalyzes re-folding. Indeed, we found that GRP78, over a range of concentrations, yielded an increase in A_620_ as evidence of liberated free phosphate (Pi; Fig. 4A). However, PST (at 1 or 10 µM) displayed a significant *inhibitory* effect (∼25% and ∼60% respectively) on the ATPase activity of GRP78 (Fig. 4B). The IC_50_ for this inhibition was estimated at ∼5.2 µM (Fig. 4B).

To test the effect of PST on GRP78 *expression*, we used a plasmid containing the GRP78 promoter region driving a luciferase reporter. Basal promoter activity did not change with PST, but PST substantially (by ∼32%) *inhibited* up-regulation of the GRP78 promoter during UPR activation by tunicamycin (Fig. 4C).

### PST alters glucose metabolism and insulin action

**Figure 5:**
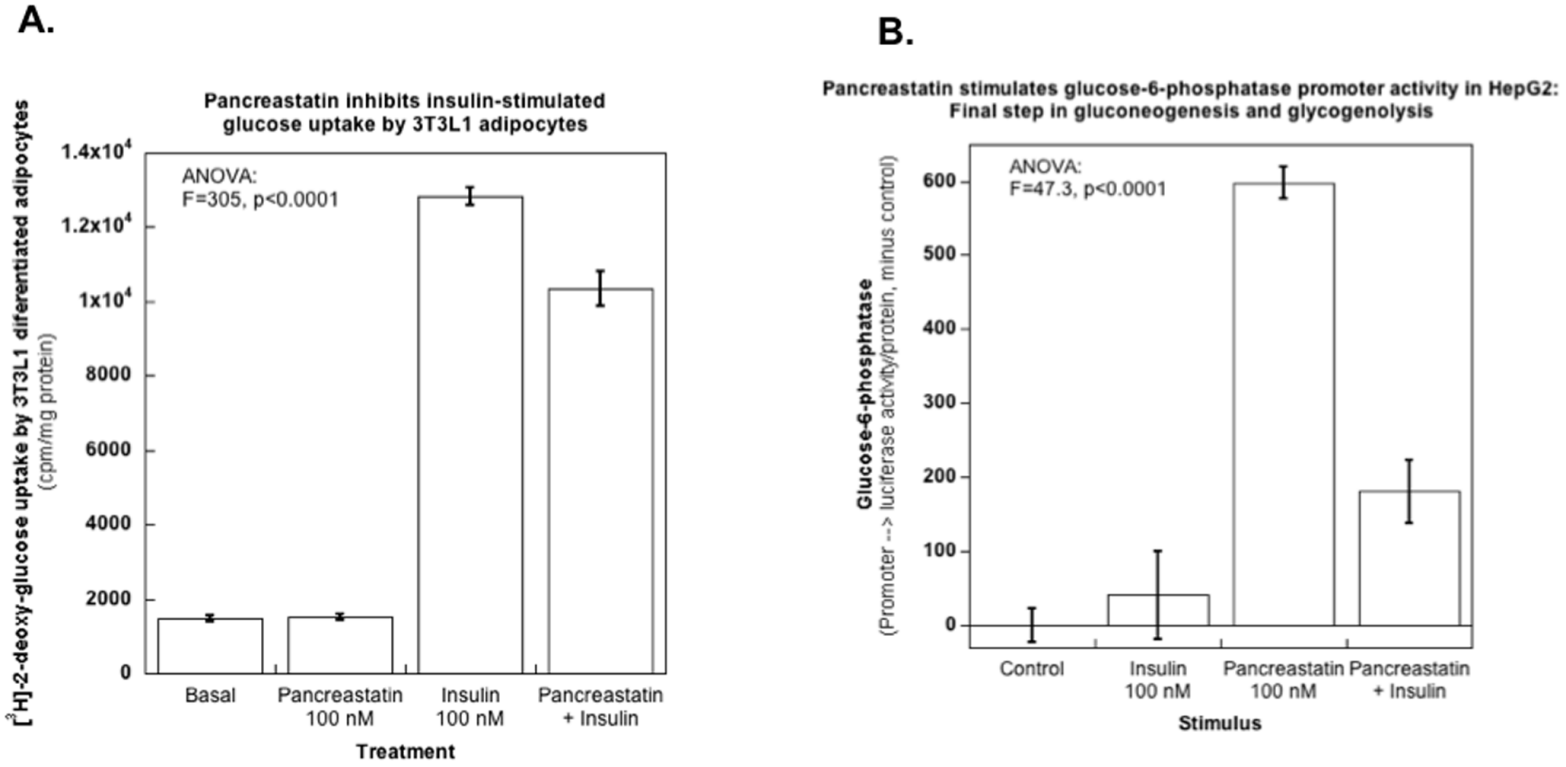
PST acts on both *uptake* and *mobilization* steps for glucose: Effects on cellular glucose metabolism and insulin action. (**A**) **PST and glucose** ***uptake***. Mouse 3T3-L1 cells were induced to differentiate into adipocytes, and 7–10 days post-differentiation glucose uptake was measured by incubating cells with 2-deoxy-[^3^H]-glucose in the presence or absence of PST (100 nM), insulin (100 nM), or insulin plus PST (100 nM each). (**B**) **Glucose** ***mobilization*** **and PST**: Effects on expression of the gluconeogenic/glycogenolytic enzyme G6Pase (glucose-6-phosphatase). Human HepG2 hepatocytes were transfected with a glucose-6-phosphatase (G6P-ase)→luciferase reporter plasmid along with a beta gal reporter plasmid as mentioned in the experimental procedures, and 5 hrs after transfection cells were treated with PST (100 nM), insulin (100 nM), or both insulin and PST (100 nM each). Cells were harvested at 18–20 hrs after transfection to measure luciferase activity, beta gal activity and protein.

In 3T3-L1 adipocytes, we found a ∼30% inhibition of insulin-stimulated glucose uptake by PST (Fig. 5A). To gain a better understanding of PST's role in glucose release, we transfected HepG2 hepatocytes with a G6Pase promoter/luciferase reporter plasmid, since G6Pase catalyzes the final step in both gluconeogenesis and glycogenolysis, resulting the formation of glucose plus free phosphate from glucose-6-phosphate. G6Pase expression was *up-regulated* ∼1.4-fold by PST (Fig. 5B). Insulin suppressed PST-induced activation of the G6Pase promoter by ∼40% (Fig. 5B).

### PST inhibition of GRP78 ATPase enzymatic activity stimulates transcription of the gluconeogenic/glycogenolytic enzyme G6Pase

**Figure 6:**
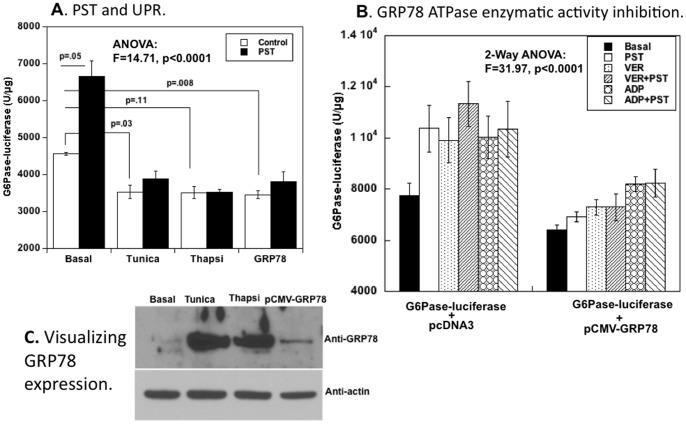
Role of the gluconeogenic/glycogenolytic enzyme G6Pase: Response to PST, GRP78, and inhibition of GRP78's ATPase enzymatic activity. (**A**) **G6Pase expression, PST, and GRP78**. Human HepG2 hepatocytes were transfected with G6P-ase→luciferase, along with pCMV-GRP78 or an empty vector (pCMV promoter without insert). 5 hrs after transfection, cells were treated with PST (100 nM) itself (versus mock), or by UPR activation (tunicamycin, 5 µg/mL; or thapsigargin, 0.3 µM). Cells were harvested at 18–20 hrs to measure luciferase activity and protein. (**B**) **G6Pase expression during ATPase enzymatic activity inhibition of GRP78**. Human HepG2 hepatocytes were transfected as in (A), and 5 hrs later were treated with the GRP78 ATPase inhibitors VER-155008 (1 µM), ADP (10 µM), or PST (100 nM). Cells were harvested at 18–20 hrs to measure luciferase activity and protein. (**C**) **GRP78 expression: Visualization by immunoblot**. Changes in GRP78 expression are shown by anti-GRP78 immunoblot (control: anti-actin), during either UPR activation (by thapsigargin 0.3 µM or tunicamycin 5 µg/mL), or exogenous over-expression (transfection of pCMV-GRP78).

Since G6Pase is involved in the final step of both gluconeogenesis and glycogenolysis, we tested the effect of GRP78 as well as UPR activation on G6Pase expression (Figure 6A). In this system, PST augmented G6Pase expression by ∼45%; during UPR activation or GRP78 addition, basal G6Pase expression declined by ∼22–24%, and the PST-induced increment was abolished. As internal controls, at the protein level, activation of the hepatocyte UPR (by tunicamycin or thapsigargin) resulted in a ∼15-fold increase in GRP78 protein expression as compared to untreated cells (Fig. 6C), while in cells co-transfected with a GRP78-expression plasmid, a moderate (∼2-fold) increase in GRP78 protein expression was observed.

To further clarify how GRP78 affects G6Pase expression, we used adenosine-derived compounds that bind to and inhibit the GRP78 ATPase domain: VER-155008 and adenosine 5′-diphosphate (ADP). With either VER-155008 or ADP addition, basal G6Pase-luciferase promoter activity rose (Fig. 6B) to a level comparable to PST activation. Again, over-expression of GRP78 reduced G6Pase promoter activity, though inhibition of GRP78 ATPase activity by PST, VER-155008 or ADP could not fully overcome the effect of GRP78 over-expression.

## Discussion

### Overview

To identify potential target proteins for PST action, we used ligand affinity chromatography with biotinylated human PST (hCgA_273–301_-amide) as “bait” on a murine liver homogenate (as “prey”), and found that PST interacts in pH-dependent fashion with GRP78. GRP78 (also known as HSPA5 or BIP) belongs to a group of heat shock proteins (or chaperones) playing a role in folding and assembly of nascent and malfolded proteins in the lumen of the endoplasmic reticulum (ER) [32].

In stressful conditions such as accumulation of misfolded proteins, the capacity of these chaperones may become inadequate, leading to a condition defined as “ER stress”. The cellular response to ER stress, referred to as the Unfolded Protein Response (UPR), activates signaling pathways from three ER stress sensors: Inositol-Requiring protein 1 (IRE1α), PKR-like ER Kinase (PERK) and Activation Transcription Factor 6 (ATF6). The actions of these signaling cascades serve to reduce ER stress through induction of chaperones and attenuation of protein translation.

Overall GRP78 balance within the cell is thus critical for secretory pathway homeostasis (the adaptive UPR) and avoidance of programmed cell death (the pro-apoptotic UPR) [33]. GRP78 has been associated with dysglycemic disease states as well as glucose metabolism in several ways [34]–[36]. Previous studies have shown that ER stress may impair insulin action in adipocytes and hepatocytes, as well as reduce insulin secretion from pancreatic islet beta cells; in such settings, a relative deficit in the UPR may play a causal role in the development of type 2 diabetes [37]–[39]. Indeed, haploinsufficiency of GRP78 attenuates diet-induced obesity and insulin resistance in the GRP78(+/−) heterozygote mouse [40].

### ER stress and induction of the UPR

Induction of GRP78 biosynthesis by ER stress is mediated by multiple copies of the ER stress response element (ERSE) [41], [42], through which both ATF6 and XBP1 activate GRP78 transcription. Insulin and IGF-1 can regulate GRP78 expression and augment the adaptive capacity of the UPR under ER stress conditions [43]–[48]. Although GRP78 expression is a downstream target of insulin [43], recent evidence suggests that GRP78 and the other adaptive ER chaperone proteins regulate organismal insulin sensitivity and glucose homeostasis [35], [36], [49], [50], in addition to protecting cells during acute stress.

Genetic ablation of CHGA in the mouse leads to global over-expression of not only GRP78 (Fig. 2A) but also other heat shock transcripts in liver (Fig. 3A), while PST itself down-regulates GRP78 activity and synthesis (Fig. 4B & C). The UPR may subserve both adaptive (stress-survival) and pro-apoptotic (programmed cell death) outcomes [51]; within the UPR, heat shock proteins (HSPs) are largely adaptive (i.e., pro-survival in the face of stress). Pro-apoptotic transcripts were globally down-regulated in CHGA(−/−) mouse liver (Fig. 3B). CHGA(−/−) mice also display improved glycemia with enhanced insulin sensitivity *in vivo* [23]. The observation that activation of the adaptive UPR leads to enhanced insulin sensitivity with decreased obesity has been described previously [40].

### Role of GRP78 and its ATPase enzymatic activity

As a member of the HSP70 family, the molecular chaperone GRP78 (BIP, HSPA5) is involved in several cellular processes, including regulation of calcium homoeostasis, translocation of newly synthesized polypeptides across the endoplasmic reticulum membrane and their subsequent folding, maturation and transport. HSP70-group proteins harbor two functional domains: a ∼44 kDa amino-terminal domain possesses ATPase activity, while a carboxyl-terminal domain consists of a ∼20 kDa peptide-binding sub-domain, followed by a helical and variable ∼10 kDa carboxyl-terminal tail. GRP78 and other HSP70 proteins self-associate into multiple oligomeric forms, and the binding of an unfolded peptide substrate onto the carboxyl-terminal domain (or binding of ATP onto the amino-terminal domain) promotes depolymerization and stabilization of the GRP78 monomer [52].

GRP78 contacts a variety of proteins in the secretory pathway, such as the luminal “J” domain of the transmembrane protein MTJ1, thereby stimulating GRP78's ATPase enzymatic activity, as an energetic source for the re-folding process [53].

In contrast to such characteristic *activation* of chaperone ATPase enzymatic activity, we observed *inhibition* of GRP78's ATPase activity by PST (Fig. 4B). PST as well as other ATPase inhibitors (VER, ADP) activated G6Pase expression (Fig. 6B, left), suggesting that such GRP78 ATPase inhibition may be central to the dysglycemic effects of PST.

GRP78 over-expression *reduced* G6Pase promoter activity (Fig. 6B, right), reinforcing the diametrically opposing effects of PST and GRP78 on the final step in the gluconeogenic/glycogenolytic cascades (Fig. 6A). The reduction in G6Pase expression by GRP78 over-expression could still be partially reversed by inhibitors of GRP78 ATPase activity (PST, VER-155008, ADP; Fig. 6B, right).

### Hepatocytes, glucose, and PST

Obesity, type-2 diabetes, and the associated hepatic steatosis are metabolic disorders characterized by insulin resistance. ER stress activation in the setting of the metabolic syndrome is extensively documented, and chaperone balance may regulate insulin sensitivity and glucose homeostasis [35], [36], [49], [50].

GRP78 protein expression is decreased in liver of obese/diabetic db/db (leptin receptor-deficient) mice, suggesting that the UPR response to ER stress may be defective [54]. In the same db/db model, administration of the chemical chaperones 4-phenylbutyric acid (PBA) or TUDCA (tauroursodeoxycholic acid) reduced ER stress, with restored glucose homeostasis and improved insulin sensitivity [38].

Similarly, adenovirus-mediated short-term GRP78 *over*-expression reduced hepatic steatosis and improved insulin sensitivity in db/db mice [55]. In contrast, long-term GRP78 *under*-expression in heterozygous (+/−) GRP78 knockout mice conferred protection from obesity and insulin resistance after exposure to high-fat diet [40]; in that study, high fat diet activated other chaperones of the adaptive UPR, thereby improving ER quality control and perhaps contributing to metabolic protection.

We established insulin counter-regulatory effects of PST in 3T3-L1 adipocytes and HepG2 hepatocytes (Fig. 5A & 5B), wherein PST inhibits insulin-stimulated glucose uptake while activating the promoter of the gluconeogenic/glycogenolytic enzyme G6Pase. G6Pase expression is ultimately regulated by a number of hormones and metabolites, including insulin, glucagon, glucose, and glucocorticoids [56].

### Advantages and limitations of this study

Here we report a novel interacting partner of the dysglycemic PST fragment of CHGA, identifying the UPR chaperone GRP78 (HSPA5, BIP). The binding was pH-dependent, verified by numerous diagnostic peptides on LC-tandem-MS/MS (Figure 1, Table 1, Table S1), and had functional consequences in inhibition of GRP78's ATPase enzymatic activity (Figure 4B).

While previous reports have emphasized potential signaling pathways for PST [23], we can now propose a specific molecule as the PST target that initiates its cascade of metabolic events. Future studies regarding PST's interaction with GRP78 and subsequent cell signaling should benefit our understanding of the pathophysiology of insulin resistance.

Nonetheless, unanswered questions remain. For example, exactly how (and by what pathway) does PST's inhibition of GRP78's ATPase enzymatic activity eventuate in G6Pase activation and consequent dysglycemia?

How might circulating PST contact GRP78, which typically exhibits an intra-cellular location within the ER? A cell surface (plasma membrane) form of GRP78 has been described, especially on cells undergoing the ER stress response as well as on cancer cells [57], [58]. Indeed, cell surface GRP78 has been demonstrated to act as a receptor, and several peptides up-regulated in cancer can bind to cell surface GRP78 and thereby influence cell proliferation [59]–[63]. Some studies have implicated AKT activation in cell surface GRP78 signaling [61], [63], a downstream pathway of insulin signaling. We have not explored a specific role of cell surface GRP78. The GRP78va [31] splice variant isoform of GRP78 is another potential target of PST, but the GRP78va isoform seems to be predominantly cytosolic, and analysis of the identified GRP78 peptide fragments (Figure 1B, Table S1) indicated that the PST binding partner was GRP78 itself (rather than GRP78va).

Is there more than one PST receptor? Previous studies on circulating pancreatatin indicate a concentration of ∼5–30 pM in humans [64], with effects demonstrable on forearm glucose uptake at ∼200 nM, and glucose uptake inhibitory effects on adipocytes with IC_50_ values of ∼0.6 nM. Gayen et al [23] also found effects of PST on hepatocytes and adipocytes at 10–100 nM PST. By contrast, we found here that PST inhibited GRP78's ATPase enzymatic activity at IC*_50_*∼5.2 µM (Fig. 4). Thus, it is conceivable that additional, higher-affinity PST receptors exist. However, we detected no evidence of a PST GPCR during ligand-affinity experiments (Table 1).

### Conclusions and perspectives

**Figure 7:**
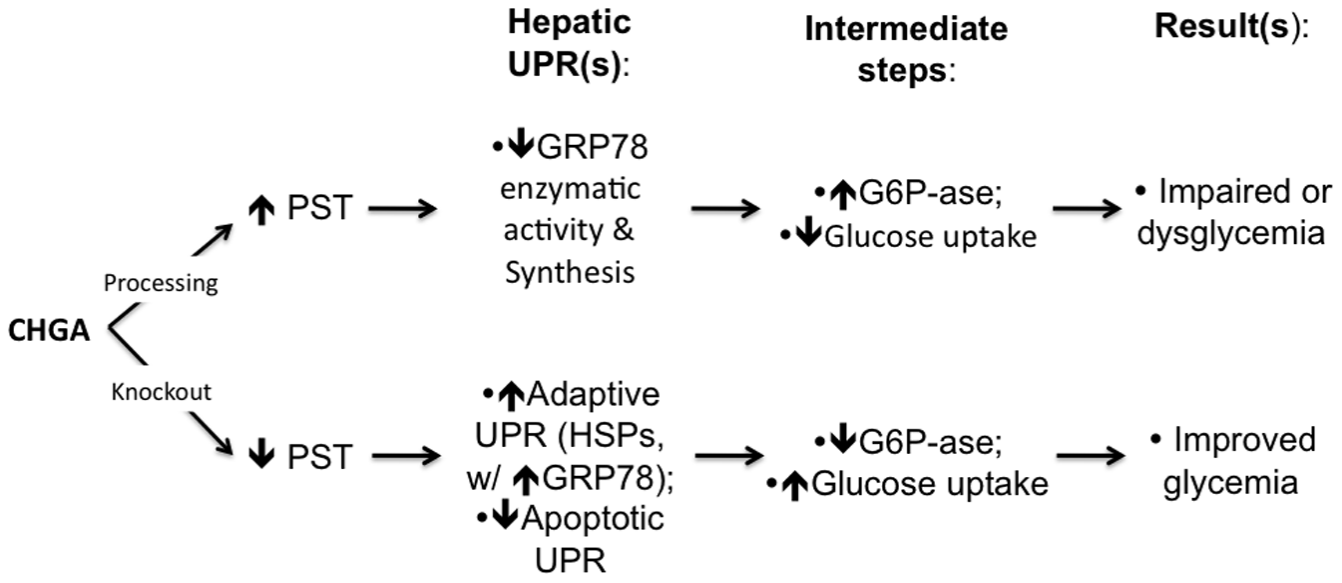
The CHGA dysglycemic fragment pancreastatin (PST): Interactions with GRP78 and the hepatic UPR(s) to achieve metabolic effects. The diagram is presented as a hypothetical schema integrating the experimental results.

Since CHGA is released along with catecholamines during sympatho-chromaffin activity, especially under stressful conditions, the PST fragment of CHGA may thus play a role in the pathophysiology of organismal stress by regulating the supply of glucose (and hence energy) to multiple tissues. Under resting circumstances, such actions on glucose metabolism may prove to be deleterious. In Fig. 7, we present a schema integrating our experimental results into a model for PST action via GRP78 and the ER stress pathway. We propose that identification of a novel binding target for ∼µM range PST concentrations may advance our understanding of glycemic control, and provide a new avenue of investigation into pathways subserving dysglycemia.

## Supporting Information

Table S1 **List of peptides identified during MS/MS by Protein Pilot software**. Sheet 1 for GRP78 (HSPA5, BIP), sheet 2 for stress 70 protein (GRP75, HSPA9) and sheet 3 for heat shock cognate 71 kDa protein (HSPA8). N represents the rank of the specified protein relative to all other proteins detected. A measure of the protein confidence for a detected protein in terms of ProtScore (either unused or total) and three different measures of % coverage [% Cov, % Cov (50) and % Cov (95)] are shown.
(XLSX)
